# Atypical B12 Deficiency with Nonresolving Paraesthesia

**DOI:** 10.1155/2013/823842

**Published:** 2013-11-18

**Authors:** S. Haider, N. Ahmad, E. J. Anaissie, N. Abdel Karim

**Affiliations:** ^1^Department of Internal Medicine, University of Cincinnati College of Medicine, Cincinnati, OH 45267, USA; ^2^Division of Hematology and Oncology, University of Cincinnati College of Medicine, Cincinnati, OH 45267, USA

## Abstract

Vitamin B12 deficiency can present with various hematological, gastrointestinal and neurological manifestations. We report a case of elderly female who presented with neuropathy and vitamin B12 deficiency where the final work-up revealed polyneuropathy, organomegaly, endocrinopathy, monoclonal gammopathy, and skin changes (POEMS). This case suggests that, although POEMS syndrome is a rare entity, it can present with vitamin-B12 deficiency and thus specific work up for early diagnosis of POEMS should be considered in patients with B12 deficiency unresponsive to therapy.

## 1. Introduction

Patients with vitamin B12 deficiency usually present with severe macrocytic anemia (mean corpuscular volume (MCV) > 100 fL) with or without varying neurologic disturbances. In large surveys in the United States and the United Kingdom, about 6% of those aged ≥60 years are vitamin B-12 deficient (plasma vitamin B-12 < 148 pmol/L), with the prevalence of deficiency increasing with age. In one series of 173 patients with vitamin B12 deficiency, 74% presented with neurologic symptoms [1]. Rare patients are unresponsive to the adequate B12 replacement therapy. This is the first case report of a patient with B12 deficiency unresponsive to replacement therapy but also had another underlying syndrome.

## 2. Case Presentation

Our case report describes 68-year-old female patient who presented initially with neuropathy attributed to vitamin B12 deficiency. Unfortunately, she developed multitude of symptoms including numbness and tingling in both feet, lack of normal sensation up to mid-calf, worsening balance and new onset red papules around shoulder, lower abdomen and back, and unintentional weight loss (15 pounds in 6 months). She was diagnosed with vitamin B12 deficiency and treated with weekly doses of vitamin B12. Laboratory work-up showed serum M (SM) 0.1 g/dL, immunoglobulin G monoclonal protein with lambda light chain specificity on immunofixation, lambda light chain of 85, 24 hour urine protein was normal at 135.7, urine protein electrophoresis was normal. Bone marrow revealed hyper cellular marrow with 50% cellularity and 10% plasma cells on biopsy. Human immunodeficiency virus and human herpes virus 8 were negative. Skeletal survey showed expansile lytic lesion at third lumbar vertebra (L3). PET showed expansile L3 lytic lesion with SUV of 14.8, enlarged left periaortic lymph node with SUV of 9.4. MRI revealed large, lobulated lytic expansile lesion/mass involving the left L3. US abdomen did not show hepatomegaly, splenomegaly, or abdominal free fluid. Electromyography revealed demyelinating sensorimotor peripheral neuropathy. Analysis of cerebrospinal fluid (CSF) showed glucose 58, protein 81, RBCs 131, nucleated cells 12, neutrophils 18%, lymphocytes 74%, and macrophage 8%. Fat pad biopsy on two occasions was negative for Congo red stains and amyloidosis. Right shoulder excision showed cherry angioma. Differential diagnosis included monoclonal gammopathy of undetermined significance (MGUS) [2], solitary plasmacytoma, multiple myeloma, Waldenstrom macroglobulinemia, primary amyloidosis, and cryoglobulinemia. Our patient did not fulfill the Mayo Clinic criteria for the diagnosis of POEMS which in addition to demyelinating peripheral neuropathy and monoclonal protein also require at least one of the following three major criteria: osteosclerotic myeloma, Castleman's disease, or elevated serum or plasma serum vascular endothelial growth factor (VEGF) level. In this case, both features were lacking and her serum VEGF level was normal. Castleman's disease required a lymph node biopsy for diagnosis and the only lymphadenopathy present was difficult to biopsy in a patient on anticoagulation. The osteosclerotic lesions in POEMS could be difficult to detect in her large L3 lytic lesion. She had five minor criteria for POEMS: organomegaly (large periaortic lymph node 3.6 × 2.4 × 2.8 cm on PET with high FDG uptake), elevated serum parathyroid hormone, cherry angiomas, erythrocytosis, hemoglobin 16.9 g/dL with decreased EPO levels in absence of myeloproliferative disorder, Jak 2 mutation and BCR-ABL, and thrombocytosis (platelets of 445,000/mcL). Bone marrow biopsy findings highly suggestive of POEMS and showed monoclonal lambda restricted plasma cells, multiple reactive lymphoid aggregates and megakaryocyte hyperplasia (mild). Unexplained weight loss (~15 pounds over the past several weeks). There were no CRAB features of multiple myeloma and fat pad biopsy was negative for amyloidosis.

Patient was treated with radiation to the L3 lytic lesion using 50 Gy with complete pain resolution in six weeks. She subsequently received systemic therapy with cyclophosphamide 600 mg weekly and decadron 12 mg weekly for six weeks. Bortezomib  0.7 mg/m^2^ weekly was added for four weeks. This resulted in stable SM and lambda to kappa light chain ratio. Her peripheral neuropathy was worsened. She has been maintained on IV melphalan 20 mg/m^2^ and platelets were kept >100.000. Concern with high dose melphalan and autologous stem cell transplant is thrombocytopenia and risk of bleeding.

## 3. Discussion

Polyneuropathy, organomegaly, endocrinopathy, monoclonal gammopathy, and skin changes-POEMS [3, 4] is a rare disease with prevalence of 1 in 1000000. It can present in a variety of manners and has different diagnostic criteria. Mayo Clinic Criteria for the diagnosis of POEMS syndrome require the presence of at least three major criteria (polyneuropathy, monoclonal plasma cell disorder, and any one of the following three: osteosclerotic myeloma, Castleman's disease, or elevated serum or plasma VEGF levels at least three to four times the upper limit of normal), along with the presence of at least one of the six minor criteria organomegaly (splenomegaly, hepatomegaly, or lymphadenopathy), extravascular volume overload (edema, pleural effusion, or ascites), endocrinopathy (adrenal, thyroid, pituitary, gonadal, parathyroid, pancreatic), Skin changes (hyperpigmentation, hypertrichosis, glomeruloid hemangiomata, and plethora, acrocyanosis, flushing, and white nails) papilledema, thrombocytosis/polycythemia. Cause unknown, likely secondary to increased VEGF [5], mostly 5 to 10 fold more than controls [6]. Plasma VEGF level >200 pg/mL had a sensitivity and specificity of 68 and 95 percent, respectively, in support of a diagnosis of POEMS syndrome. VEGF can be followed to evaluate response to therapy [7]. Patients of POEMS have higher levels of IL-1B, TNF-alpha and IL-6 as compared to multiple myeloma. Symptoms begin as distal symmetric sensory changes in nerves of feet. Motor changes follows and cranial nerves are spared. CSF proteins are elevated with normal white count. Biopsy of the sural nerve usually shows both axonal degeneration and demyelination; severe endoneurial edema may also be seen. Skin changes [8, 9] (hyperpigmentation, hypertrichosis, acrocyanosis, plethora, and hemangioma/telangiectasia) are noted in two thirds of patients. Osteosclerotic bone lesions appeared in conventional radiographs in 97 percent of patients in the Mayo Clinic study. The pelvis, spine, ribs, and proximal extremities were most often involved. Castleman's disease (giant cell lymph node hyperplasia, and angiofollicular lymph node hyperplasia) and POEMS syndrome have been frequently associated. 15% of patients with POEMS have Castleman's disease. Antibodies to human herpes virus 8 (HHV 8) are frequently reported in Castleman's disease. Vascular events most often consisted of cerebral infarction, myocardial infarction, or Budd-Chiari syndrome. The course of POEMS syndrome is chronic; patients survive three times longer compared with multiple myeloma. The natural history is one of progressive peripheral neuropathy until the patient is bedridden [10]. Death usually occurs from inanition or a terminal bronchopneumonia. Overall median survival was 13.7 years in the Mayo Clinic series, while those with clubbing or extravascular volume overload had median survival of 2.6 and 6.6 years, respectively. There is currently insufficient evidence regarding the treatment options for POEMS syndrome on which to base practice [11]. Supportive care as required for neurological and respiratory problems—for example, physiotherapy, occupational therapy, respiratory support or multidisciplinary care.

Radiotherapy—if the osteosclerotic lesions are single or restricted to a limited area. If osteosclerotic lesions are widespread—chemotherapy or autologous stem cell transplantation [12].

Possible treatments (from small series and case reports) are

 melphalan and prednisolone;
 cyclophosphamide [13] ± prednisolone;
 corticosteroids alone, which may help stabilize the disease;
 high-dose chemotherapy with stem cell transplantation [14], although this carries significant risks [15];
 lenalidomide or thalidomide. There are concerns that thalidomide also causes peripheral neuropathy and fluid retention. Lenalidomide was helpful in one reported case [16];
 bevacizumab therapy, which has been reported as beneficial in some cases [17–19];

## 4. Conclusion

POEMS should be suspected in a patient presenting with neuropathy, in the setting of vitamin B12 deficiency unresponsive to B12 replacement therapy. It can present with variety of symptoms due to the involvement of multiple organs. It is critical to look for signs of other organ systems involved, specific lab testing including VEGF levels to avoid delay in accurate diagnosis and treatment.
